# Di-μ-bromido-bis­({2-[(4,6-dimethyl­pyrimidin-2-yl)disulfan­yl]-4,6-dimethyl­pyrimidine-κ^2^ *N* ^1^,*S* ^2^}copper(I))

**DOI:** 10.1107/S1600536812016315

**Published:** 2012-04-21

**Authors:** Ruthairat Nimthong, Chaveng Pakawatchai, Yupa Wattanakanjana

**Affiliations:** a Department of Chemistry and Center for Innovation in Chemistry, Faculty of Science, Prince of Songkla University, Hat Yai, Songkhla 90112, Thailand; b Department of Chemistry, Faculty of Science, Prince of Songkla University, Hat Yai 90112, Thailand

## Abstract

The title dinuclear complex, [Cu_2_Br_2_(C_12_H_14_N_4_S_2_)_2_], is located about an inversion center. The Cu^I^ ion is coordinated in a distorted tetra­hedral geometry by two bridging Br atoms in addition to an N and an S atom from the 2-[(4,6-dimethyl­pyrimidin-2-yl)disulfan­yl]-4,6-dimethyl­pyrimidine ligand. In the crystal, π–π stacking inter­actions are observed with a centroid–centroid distance of 3.590 (2) Å.

## Related literature

For potential applications of heterocyclic thio­amides and their metal complexes, see: Battistuzzi & Peyronel (1981 ▶); Holm & Solomon (1996 ▶); Cox *et al.* (2006 ▶); Falcomer *et al.* (2006 ▶); Sevier & Kaiser (2006 ▶); Saxena *et al.* (2009 ▶). For related structures, see: Lemos *et al.* (2001 ▶); Aslanidis *et al.* (2004 ▶); Freeman *et al.* (2008 ▶).
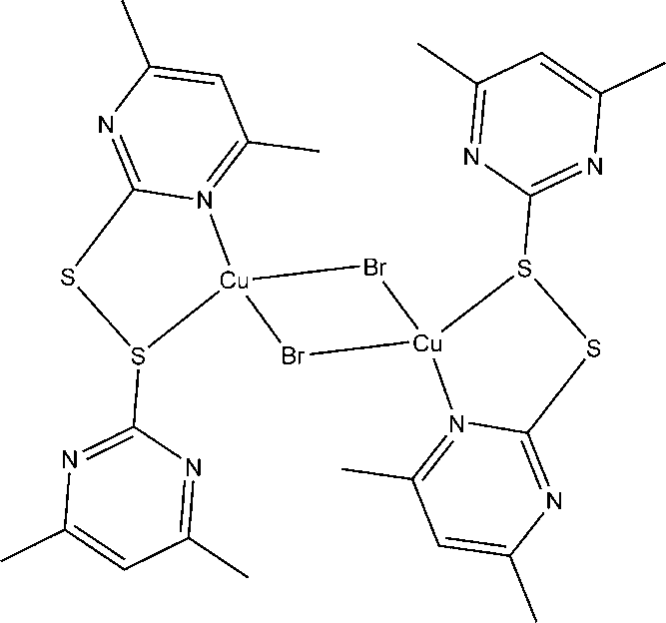


## Experimental

### 

#### Crystal data

[Cu_2_Br_2_(C_12_H_14_N_4_S_2_)_2_]
*M* *_r_* = 843.68
Monoclinic, 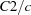

*a* = 15.3351 (7) Å
*b* = 15.3898 (7) Å
*c* = 14.3398 (7) Å
β = 109.178 (1)°
*V* = 3196.4 (3) Å^3^
*Z* = 4
Mo *K*α radiation
μ = 4.12 mm^−1^
*T* = 293 K
0.21 × 0.18 × 0.10 mm

#### Data collection

Bruker SMART CCD diffractometer
Absorption correction: integration (*SADABS*; Bruker, 2003 ▶) *T*_min_ = 0.425, *T*_max_ = 0.662
12339 measured reflections
2732 independent reflections
2344 reflections with *I* > 2σ(*I*)
*R*_int_ = 0.024

#### Refinement

*R*[*F* ^2^ > 2σ(*F* ^2^)] = 0.028
*wR*(*F* ^2^) = 0.077
*S* = 1.04
2732 reflections
181 parameters
55 restraints
H-atom parameters constrained
Δρ_max_ = 0.37 e Å^−3^
Δρ_min_ = −0.31 e Å^−3^

### 

Data collection: *SMART* (Bruker, 1998 ▶); cell refinement: *SAINT* (Bruker, 2003 ▶); data reduction: *SAINT*; program(s) used to solve structure: *SHELXS97* (Sheldrick, 2008 ▶); program(s) used to refine structure: *SHELXL97* (Sheldrick, 2008 ▶); molecular graphics: *SHELXTL* (Sheldrick, 2008 ▶); software used to prepare material for publication: *SHELXL97* and *publCIF* (Westrip, 2010 ▶).

## Supplementary Material

Crystal structure: contains datablock(s) I, global. DOI: 10.1107/S1600536812016315/lh5449sup1.cif

Structure factors: contains datablock(s) I. DOI: 10.1107/S1600536812016315/lh5449Isup2.hkl

Additional supplementary materials:  crystallographic information; 3D view; checkCIF report

## Figures and Tables

**Fig. 1.**
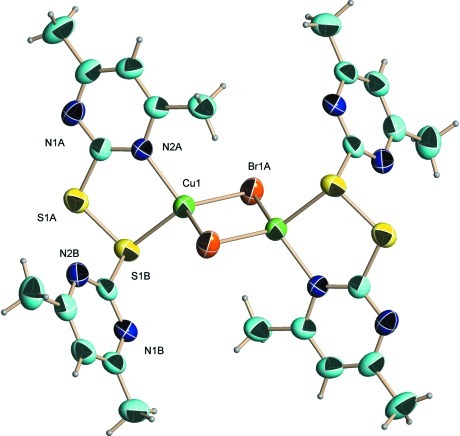
The molecular structure with displacement ellipsoids drawn at the 50% probability level. Unlabeled atoms are related by (-x+1/2, -y+1/2, -z+1).

**Fig. 2.**
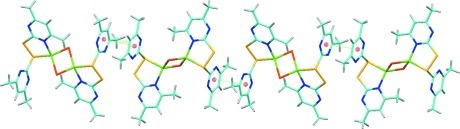
Part of the crystal structure with π–π stacking interactions shown as dashed lines.

**Fig. 3.**
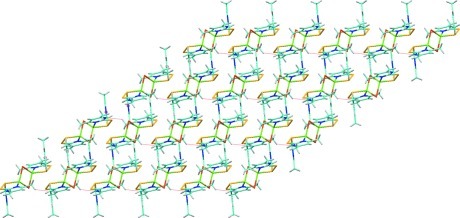
Part of the crystal structure with weak C—H···N hydrogen bonds shown as dashed lines.

## supplementary crystallographic information

### Comment

The studies of cooordination multidentate ligands such as heterocyclic thioamides, in complexes of closed-shell d^10^ metal ions, have been shown attention from a number of researchers (Saxena *et al.*, 2009; Cox *et al.*, 2006; Falcomer *et al.*, 2006) because of their interesting biochemical properties and presence in active sites of many metalloproteins (Holm & Solomon, 1996; Battistuzzi & Peyronel, 1981). Particularly, the formation of disulfide bonds is an essential step in the folding and assembly of the extracellular domains of many membrane and secreted proteins which are important features of the structure of many proteins (Sevier & Kaiser, 2006).

The molecular structure of the title compound is shown in Fig. 1. The complex is dinuclear in which the Cu^I^ ions adopt distorted tetrahedral geometries. There is a binuclear *µ*,*µ*'-dibromobridged CuBr_2_Cu core. The Cu—S and Cu—N distances are similar to those reported for other thioamide containing complexes (Aslanidis *et al.*, 2004; Lemos *et al.*, 2001) and the disulfide bond distances is shorter than that reported in a related compound with a disulfide bond (Freeman *et al.*, 2008). The 'bite' angle S—Cu—N angle is 90.77 (7)°. The molecule lies on a crystallographic inversion center which is at the center of the CuBr_2_Cu core with a Cu···Cu separation of 2.7802 (7) Å. This value is close the sum of the van der Waals radii for two Cu atoms (2.8 Å). In the crystal π–π stacking interactions with a centroid to centroid distance of 3.590 (2) Å are observed (Fig. 2). In addition, fairly short C(*sp^3^*)—H···N intermolecular distances (H···N = 2.67 Å, C(*sp^3^*)—N = 3.41 Å and C(*sp^3^*)—H···N = 134.2°) are observed (Fig. 3).

### Experimental

4,6-Dimethyl-2-pyrimidinethiol, dmpymtH, (0.07 g, 0.50 mmol) was dissolved in 30 cm^3^ of methanol at 343-348K. CuBr (0.1 g, 0.70 mmol) was added and the mixture was stirred for 5 h. The resulting clear solution was filtered off and left to evaporate at room temperature. The crystalline complex, which was deposited upon standing for several days, was filtered off and dried *in vacuo* (yield 75%).

### Refinement

The H atoms bonded to C atoms were constrained with a riding model of C—H = 0.93–0.96 Å and with *U*_iso_(H) = 1.2*U*_eq_(C). The DELU instruction in SHELXL (Sheldrick, 2008) was used without any further parameters. This sets up 'rigid bond' restraints for all non-hydrogen atom. The dafault standard deviation values are 0.01 and 0.01. This appears to have little effect but it does affect the no of restraints (55) listed in the CIF.

### Crystal data

**Table:**

[Cu_2_Br_2_(C_12_H_14_N_4_S_2_)_2_]	*F*(000) = 1680
*M**_r_* = 843.68	*D*_x_ = 1.753 Mg m^−^^3^
Monoclinic, *C*2/*c*	Mo *K*α radiation, λ = 0.71073 Å
Hall symbol: -C 2yc	Cell parameters from 16645 reflections
*a* = 15.3351 (7) Å	θ = 1.9–24.7°
*b* = 15.3898 (7) Å	µ = 4.12 mm^−^^1^
*c* = 14.3398 (7) Å	*T* = 293 K
β = 109.178 (1)°	Plate, colorless
*V* = 3196.4 (3) Å^3^	0.21 × 0.18 × 0.10 mm
*Z* = 4	

### Data collection

**Table:**

Bruker SMART CCD diffractometer	2732 independent reflections
Radiation source: fine-focus sealed tube	2344 reflections with *I* > 2σ(*I*)
Graphite monochromator	*R*_int_ = 0.024
φ and ω scans	θ_max_ = 24.7°, θ_min_ = 1.9°
Absorption correction: integration (*SADABS*; Bruker, 2003)	*h* = −18→18
*T*_min_ = 0.425, *T*_max_ = 0.662	*k* = −18→17
12339 measured reflections	*l* = −16→16

### Refinement

**Table:**

Refinement on *F*^2^	Primary atom site location: structure-invariant direct methods
Least-squares matrix: full	Secondary atom site location: difference Fourier map
*R*[*F*^2^ > 2σ(*F*^2^)] = 0.028	Hydrogen site location: inferred from neighbouring sites
*wR*(*F*^2^) = 0.077	H-atom parameters constrained
*S* = 1.04	*w* = 1/[σ^2^(*F*_o_^2^) + (0.0412*P*)^2^ + 3.1838*P*] where *P* = (*F*_o_^2^ + 2*F*_c_^2^)/3
2732 reflections	(Δ/σ)_max_ = 0.001
181 parameters	Δρ_max_ = 0.37 e Å^−^^3^
55 restraints	Δρ_min_ = −0.31 e Å^−^^3^

### Special details

**Table:**

Geometry. All e.s.d.'s (except the e.s.d. in the dihedral angle between two l.s. planes) are estimated using the full covariance matrix. The cell e.s.d.'s are taken into account individually in the estimation of e.s.d.'s in distances, angles and torsion angles; correlations between e.s.d.'s in cell parameters are only used when they are defined by crystal symmetry. An approximate (isotropic) treatment of cell e.s.d.'s is used for estimating e.s.d.'s involving l.s. planes.
Refinement. Refinement of *F*^2^ against ALL reflections. The weighted *R*-factor *wR* and goodness of fit *S* are based on *F*^2^, conventional *R*-factors *R* are based on *F*, with *F* set to zero for negative *F*^2^. The threshold expression of *F*^2^ > σ(*F*^2^) is used only for calculating *R*-factors(gt) *etc*. and is not relevant to the choice of reflections for refinement. *R*-factors based on *F*^2^ are statistically about twice as large as those based on *F*, and *R*- factors based on ALL data will be even larger.

### Fractional atomic coordinates and isotropic or equivalent isotropic displacement parameters (Å^2^)

**Table:**

	*x*	*y*	*z*	*U*_iso_*/*U*_eq_	
C1A	0.12116 (19)	0.06625 (19)	0.6388 (2)	0.0437 (7)	
C2A	0.0939 (2)	−0.0746 (2)	0.5935 (3)	0.0541 (8)	
C3A	0.1153 (2)	−0.0561 (2)	0.5100 (3)	0.0559 (8)	
H1A	0.1118	−0.0994	0.4637	0.067*	
C4A	0.1417 (2)	0.0265 (2)	0.4949 (2)	0.0491 (7)	
C5A	0.0690 (3)	−0.1640 (2)	0.6172 (4)	0.0806 (12)	
H2A	0.0531	−0.1622	0.6766	0.097*	
H4A	0.1206	−0.2022	0.6264	0.097*	
H3A	0.0172	−0.1849	0.5637	0.097*	
C6A	0.1667 (3)	0.0518 (3)	0.4062 (3)	0.0738 (11)	
H7A	0.1835	0.1121	0.4107	0.089*	
H5A	0.1147	0.0423	0.3477	0.089*	
H6A	0.2178	0.0173	0.4033	0.089*	
C1B	0.0927 (2)	0.3281 (2)	0.6662 (2)	0.0444 (7)	
C2B	0.0657 (2)	0.4721 (2)	0.6545 (2)	0.0535 (8)	
C3B	−0.0270 (2)	0.4533 (2)	0.6114 (2)	0.0572 (8)	
H1B	−0.0700	0.4979	0.5910	0.069*	
C4B	−0.0549 (2)	0.3680 (2)	0.5989 (2)	0.0534 (8)	
C5B	0.1020 (3)	0.5630 (2)	0.6741 (3)	0.0741 (11)	
H2B	0.1679	0.5614	0.7044	0.089*	
H4B	0.0749	0.5914	0.7174	0.089*	
H3B	0.0866	0.5943	0.6129	0.089*	
C6B	−0.1546 (2)	0.3429 (3)	0.5541 (3)	0.0743 (11)	
H5B	−0.1599	0.2807	0.5516	0.089*	
H6B	−0.1787	0.3661	0.4885	0.089*	
H7B	−0.1890	0.3658	0.5937	0.089*	
Cu1	0.20241 (3)	0.20852 (2)	0.55473 (3)	0.05128 (14)	
N1A	0.09541 (17)	−0.01146 (17)	0.65843 (19)	0.0522 (6)	
N2A	0.14596 (16)	0.09016 (15)	0.56177 (17)	0.0420 (5)	
N1B	0.12747 (18)	0.40741 (17)	0.68337 (19)	0.0515 (6)	
N2B	0.00657 (18)	0.30262 (16)	0.62617 (19)	0.0491 (6)	
S1A	0.12183 (7)	0.14055 (6)	0.73360 (6)	0.0591 (2)	
S1B	0.18435 (6)	0.24973 (5)	0.70698 (6)	0.0506 (2)	
Br1	0.13259 (2)	0.31106 (2)	0.42440 (3)	0.05795 (13)	

### Atomic displacement parameters (Å^2^)

**Table:**

	*U*^11^	*U*^22^	*U*^33^	*U*^12^	*U*^13^	*U*^23^
C1A	0.0383 (15)	0.0440 (16)	0.0466 (16)	0.0048 (12)	0.0110 (13)	0.0077 (13)
C2A	0.0375 (17)	0.0438 (17)	0.072 (2)	−0.0002 (13)	0.0056 (15)	0.0108 (16)
C3A	0.0514 (19)	0.0434 (17)	0.066 (2)	−0.0011 (14)	0.0099 (16)	−0.0083 (15)
C4A	0.0506 (18)	0.0460 (17)	0.0482 (17)	0.0027 (14)	0.0127 (14)	−0.0042 (14)
C5A	0.070 (3)	0.049 (2)	0.113 (3)	−0.0075 (18)	0.017 (2)	0.017 (2)
C6A	0.107 (3)	0.062 (2)	0.063 (2)	−0.011 (2)	0.042 (2)	−0.0166 (18)
C1B	0.0487 (17)	0.0486 (17)	0.0411 (16)	0.0076 (13)	0.0216 (13)	−0.0017 (13)
C2B	0.070 (2)	0.0468 (18)	0.0517 (18)	0.0055 (15)	0.0303 (16)	−0.0021 (14)
C3B	0.060 (2)	0.0566 (19)	0.059 (2)	0.0166 (15)	0.0256 (16)	0.0068 (16)
C4B	0.0501 (18)	0.063 (2)	0.0504 (18)	0.0081 (15)	0.0211 (14)	0.0066 (15)
C5B	0.092 (3)	0.051 (2)	0.083 (3)	0.0023 (19)	0.034 (2)	−0.0080 (19)
C6B	0.051 (2)	0.090 (3)	0.082 (3)	0.0056 (19)	0.0231 (18)	0.018 (2)
Cu1	0.0570 (3)	0.0424 (2)	0.0616 (3)	0.00037 (16)	0.0292 (2)	0.00422 (17)
N1A	0.0473 (14)	0.0483 (15)	0.0595 (16)	−0.0005 (12)	0.0157 (12)	0.0126 (13)
N2A	0.0429 (13)	0.0409 (13)	0.0426 (13)	−0.0011 (10)	0.0146 (10)	0.0018 (10)
N1B	0.0565 (16)	0.0476 (15)	0.0552 (15)	0.0014 (12)	0.0249 (12)	−0.0074 (12)
N2B	0.0477 (15)	0.0521 (15)	0.0501 (15)	0.0017 (12)	0.0197 (12)	0.0034 (12)
S1A	0.0828 (6)	0.0531 (5)	0.0510 (5)	0.0106 (4)	0.0351 (4)	0.0078 (4)
S1B	0.0495 (4)	0.0485 (5)	0.0521 (4)	0.0070 (3)	0.0142 (3)	−0.0062 (4)
Br1	0.0467 (2)	0.0548 (2)	0.0746 (3)	0.01020 (14)	0.02299 (17)	0.02122 (16)

### Geometric parameters (Å, º)

**Table:**

C1A—N1A	1.319 (4)	C2B—C3B	1.381 (5)
C1A—N2A	1.332 (4)	C2B—C5B	1.498 (5)
C1A—S1A	1.774 (3)	C3B—C4B	1.375 (5)
C2A—N1A	1.341 (4)	C3B—H1B	0.9300
C2A—C3A	1.371 (5)	C4B—N2B	1.346 (4)
C2A—C5A	1.496 (4)	C4B—C6B	1.501 (5)
C3A—C4A	1.372 (4)	C5B—H2B	0.9600
C3A—H1A	0.9300	C5B—H4B	0.9600
C4A—N2A	1.358 (4)	C5B—H3B	0.9600
C4A—C6A	1.495 (5)	C6B—H5B	0.9600
C5A—H2A	0.9600	C6B—H6B	0.9600
C5A—H4A	0.9600	C6B—H7B	0.9600
C5A—H3A	0.9600	Cu1—N2A	2.033 (2)
C6A—H7A	0.9600	Cu1—S1B	2.3754 (9)
C6A—H5A	0.9600	Cu1—Br1	2.4114 (5)
C6A—H6A	0.9600	Cu1—Br1^i^	2.4669 (5)
C1B—N2B	1.315 (4)	Cu1—Cu1^i^	2.7801 (7)
C1B—N1B	1.323 (4)	S1A—S1B	2.0318 (13)
C1B—S1B	1.798 (3)	Br1—Cu1^i^	2.4668 (5)
C2B—N1B	1.342 (4)		
			
N1A—C1A—N2A	127.9 (3)	C3B—C4B—C6B	122.1 (3)
N1A—C1A—S1A	110.3 (2)	C2B—C5B—H2B	109.5
N2A—C1A—S1A	121.7 (2)	C2B—C5B—H4B	109.5
N1A—C2A—C3A	120.0 (3)	H2B—C5B—H4B	109.5
N1A—C2A—C5A	117.2 (3)	C2B—C5B—H3B	109.5
C3A—C2A—C5A	122.8 (3)	H2B—C5B—H3B	109.5
C2A—C3A—C4A	119.9 (3)	H4B—C5B—H3B	109.5
C2A—C3A—H1A	120.0	C4B—C6B—H5B	109.5
C4A—C3A—H1A	120.0	C4B—C6B—H6B	109.5
N2A—C4A—C3A	120.3 (3)	H5B—C6B—H6B	109.5
N2A—C4A—C6A	116.5 (3)	C4B—C6B—H7B	109.5
C3A—C4A—C6A	123.2 (3)	H5B—C6B—H7B	109.5
C2A—C5A—H2A	109.5	H6B—C6B—H7B	109.5
C2A—C5A—H4A	109.5	N2A—Cu1—S1B	90.77 (7)
H2A—C5A—H4A	109.5	N2A—Cu1—Br1	122.47 (7)
C2A—C5A—H3A	109.5	S1B—Cu1—Br1	112.43 (3)
H2A—C5A—H3A	109.5	N2A—Cu1—Br1^i^	108.72 (7)
H4A—C5A—H3A	109.5	S1B—Cu1—Br1^i^	110.22 (3)
C4A—C6A—H7A	109.5	Br1—Cu1—Br1^i^	110.523 (16)
C4A—C6A—H5A	109.5	N2A—Cu1—Cu1^i^	138.63 (7)
H7A—C6A—H5A	109.5	S1B—Cu1—Cu1^i^	129.62 (3)
C4A—C6A—H6A	109.5	Br1—Cu1—Cu1^i^	56.200 (16)
H7A—C6A—H6A	109.5	Br1^i^—Cu1—Cu1^i^	54.323 (15)
H5A—C6A—H6A	109.5	C1A—N1A—C2A	116.6 (3)
N2B—C1B—N1B	129.9 (3)	C1A—N2A—C4A	115.2 (3)
N2B—C1B—S1B	120.5 (2)	C1A—N2A—Cu1	122.05 (19)
N1B—C1B—S1B	109.5 (2)	C4A—N2A—Cu1	122.3 (2)
N1B—C2B—C3B	120.1 (3)	C1B—N1B—C2B	115.2 (3)
N1B—C2B—C5B	116.9 (3)	C1B—N2B—C4B	114.3 (3)
C3B—C2B—C5B	123.0 (3)	C1A—S1A—S1B	105.86 (11)
C4B—C3B—C2B	119.3 (3)	C1B—S1B—S1A	104.42 (11)
C4B—C3B—H1B	120.4	C1B—S1B—Cu1	101.24 (10)
C2B—C3B—H1B	120.4	S1A—S1B—Cu1	99.01 (4)
N2B—C4B—C3B	121.2 (3)	Cu1—Br1—Cu1^i^	69.476 (16)
N2B—C4B—C6B	116.7 (3)		

Symmetry code: (i) −*x*+1/2, −*y*+1/2, −*z*+1.
